# 5-Cyclo­hexyl-2-(4-fluoro­phen­yl)-3-isopropyl­sulfonyl-1-benzofuran

**DOI:** 10.1107/S160053681101097X

**Published:** 2011-03-31

**Authors:** Hong Dae Choi, Pil Ja Seo, Byeng Wha Son, Uk Lee

**Affiliations:** a Department of Chemistry, Dongeui University, San 24 Kaya-dong Busanjin-gu, Busan 614-714, Republic of Korea; b Department of Chemistry, Pukyong National University, 599-1 Daeyeon 3-dong, Nam-gu, Busan 608-737, Republic of Korea

## Abstract

In the title compound, C_23_H_25_FO_2_S, the cyclo­hexyl ring adopts a chair conformation. The 4-fluoro­phenyl ring makes a dihedral angle of 50.74 (4)° with the mean plane of the benzofuran fragment. In the crystal, mol­ecules are linked by inter­molecular C—H⋯π inter­actions.

## Related literature

For the biological activity of benzofuran compounds, see: Aslam *et al.* (2009 ▶); Galal *et al.* (2009 ▶); Khan *et al.* (2005 ▶). For natural products with benzofuran rings, see: Akgul & Anil (2003 ▶); Soekamto *et al.* (2003 ▶). For structural studies of related 2-aryl-5-cyclo­hexyl-3-methyl­sulfinyl-1-benzofuran derivatives, see: Choi *et al.* (2011*a* ▶,*b* ▶).
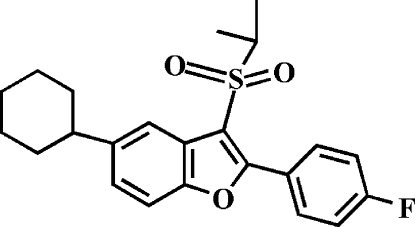


## Experimental

### 

#### Crystal data

C_23_H_25_FO_3_S
*M* *_r_* = 400.49
Monoclinic, 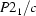

*a* = 16.2065 (3) Å
*b* = 8.4993 (2) Å
*c* = 15.4031 (3) Å
β = 110.411 (1)°
*V* = 1988.47 (7) Å^3^
*Z* = 4
Mo *K*α radiation
μ = 0.19 mm^−1^
*T* = 173 K
0.35 × 0.25 × 0.16 mm

#### Data collection

Bruker SMART APEXII CCD diffractometer
Absorption correction: multi-scan (*SADABS*; Bruker, 2009 ▶) *T*_min_ = 0.935, *T*_max_ = 0.969
34421 measured reflections
4536 independent reflections
3857 reflections with *I* > 2σ(*I*)
*R*_int_ = 0.040

#### Refinement

*R*[*F* ^2^ > 2σ(*F* ^2^)] = 0.041
*wR*(*F* ^2^) = 0.118
*S* = 1.05
4536 reflections
255 parameters
H-atom parameters constrained
Δρ_max_ = 0.43 e Å^−3^
Δρ_min_ = −0.43 e Å^−3^

### 

Data collection: *APEX2* (Bruker, 2009 ▶); cell refinement: *SAINT* (Bruker, 2009 ▶); data reduction: *SAINT*; program(s) used to solve structure: *SHELXS97* (Sheldrick, 2008 ▶); program(s) used to refine structure: *SHELXL97* (Sheldrick, 2008 ▶); molecular graphics: *ORTEP-3* (Farrugia, 1997 ▶) and *DIAMOND* (Brandenburg, 1998 ▶); software used to prepare material for publication: *SHELXL97*.

## Supplementary Material

Crystal structure: contains datablocks global, I. DOI: 10.1107/S160053681101097X/ez2240sup1.cif

Structure factors: contains datablocks I. DOI: 10.1107/S160053681101097X/ez2240Isup2.hkl

Additional supplementary materials:  crystallographic information; 3D view; checkCIF report

## Figures and Tables

**Table 1:** Hydrogen-bond geometry (Å, °) *Cg* is the centroid of the C2–C7 benzene ring.

*D*—H⋯*A*	*D*—H	H⋯*A*	*D*⋯*A*	*D*—H⋯*A*
C14—H14*A*⋯*Cg*^i^	0.99	2.68	3.667 (2)	172

Symmetry code: (i) 
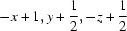
.

**Fig. 1.**
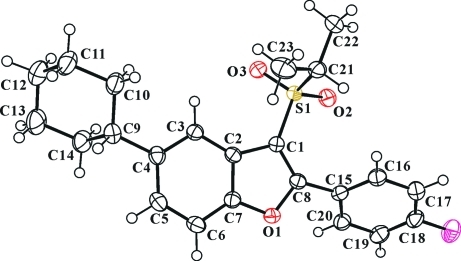
The molecular structure of the title compound with the atom numbering scheme. Displacement ellipsoids are drawn at the 50% probability level. H atoms are presented as a small spheres of arbitrary radius.

## supplementary crystallographic information

### Comment

Many compounds having a benzofuran skeleton exhibit potent biological properties such as antibacterial, antifungal, antitumor, antiviral and antimicrobial activities (Aslam *et al.*, 2009, Galal *et al.*, 2009, Khan *et al.*, 2005). These compounds occur in a wide range of natural products (Akgul & Anil, 2003; Soekamto *et al.*, 2003). As a part of our ongoing study of the substituent effect on the solid state structures of 2-aryl-5-cyclohexyl-3-methylsulfinyl-1-benzofuran analogues (Choi *et al.*, 2011**a*, b*), we report herein on the molecular and crystal structures of the title compound.

In the title compound (Fig. 1), the benzofuran unit is essentially planar, with a mean deviation of 0.010 (1) Å from the least-squares plane defined by the nine constituent atoms. The 4-fluorophenyl ring makes a dihedral angle of 50.74 (4)° with the mean plane of the benzofuran fragment. The crystal packing  is stabilized by intermolecular C—H···π interactions between a cyclohexyl H atom and the benzene ring (Table 1; C14—H14A···Cg^i^, Cg is the centroid of the C2–C7 benzene ring).

### Experimental

77% 3-chloroperoxybenzoic acid (448 mg, 2.0 mmol) was added in small portions to a stirred solution of 5-cyclohexyl-2-(4-fluorophenyl)-3-isopropylsulfanyl-1-benzofuran (331 mg, 0.9 mmol) in dichloromethane (40 mL) at 273 K. After being stirred at room temperature for 6h, the mixture was washed with saturated sodium bicarbonate solution and the organic layer was separated, dried over magnesium sulfate, filtered and concentrated at reduced pressure. The residue was purified by column chromatography (benzene) to afford the title compound as a colorless solid [yield 73%, m.p. 417–418 K; *R*_f_ = 0.66 (benzene)]. Single crystals suitable for X-ray diffraction were prepared by slow evaporation of a solution of the title compound in ethyl acetate at room temperature.

### Refinement

All H atoms were positioned geometrically and refined using a riding model, with C—H = 0.95 Å  for aryl, 1.00 Å   for methine, 0.99 Å for methylene and 0.98 Å   for methyl H atoms, respectively. *U*_iso_(H) =1.2*U*_eq_(C)  for aryl,  methine and methylene, and 1.5*U*_eq_(C) for methyl H atoms.

### Crystal data

**Table:**

C_23_H_25_FO_3_S	*F*(000) = 848
*M**_r_* = 400.49	*D*_x_ = 1.338 Mg m^−^^3^
Monoclinic, *P*2_1_/*c*	Mo *K*α radiation, λ = 0.71073 Å
Hall symbol: -P 2ybc	Cell parameters from 9904 reflections
*a* = 16.2065 (3) Å	θ = 2.7–27.5°
*b* = 8.4993 (2) Å	µ = 0.19 mm^−^^1^
*c* = 15.4031 (3) Å	*T* = 173 K
β = 110.411 (1)°	Block, colourless
*V* = 1988.47 (7) Å^3^	0.35 × 0.25 × 0.16 mm
*Z* = 4	

### Data collection

**Table:**

Bruker SMART APEXII CCD diffractometer	4536 independent reflections
Radiation source: rotating anode	3857 reflections with *I* > 2σ(*I*)
graphite multilayer	*R*_int_ = 0.040
Detector resolution: 10.0 pixels mm^-1^	θ_max_ = 27.5°, θ_min_ = 1.3°
φ and ω scans	*h* = −21→21
Absorption correction: multi-scan (*SADABS*; Bruker, 2009)	*k* = −11→10
*T*_min_ = 0.935, *T*_max_ = 0.969	*l* = −19→19
34421 measured reflections	

### Refinement

**Table:**

Refinement on *F*^2^	Primary atom site location: structure-invariant direct methods
Least-squares matrix: full	Secondary atom site location: difference Fourier map
*R*[*F*^2^ > 2σ(*F*^2^)] = 0.041	Hydrogen site location: difference Fourier map
*wR*(*F*^2^) = 0.118	H-atom parameters constrained
*S* = 1.05	*w* = 1/[σ^2^(*F*_o_^2^) + (0.0616*P*)^2^ + 0.8589*P*] where *P* = (*F*_o_^2^ + 2*F*_c_^2^)/3
4536 reflections	(Δ/σ)_max_ < 0.001
255 parameters	Δρ_max_ = 0.43 e Å^−^^3^
0 restraints	Δρ_min_ = −0.43 e Å^−^^3^

### Special details

**Table:**

Geometry. All esds (except the esd in the dihedral angle between two l.s. planes) are estimated using the full covariance matrix. The cell esds are taken into account individually in the estimation of esds in distances, angles and torsion angles; correlations between esds in cell parameters are only used when they are defined by crystal symmetry. An approximate (isotropic) treatment of cell esds is used for estimating esds involving l.s. planes.
Refinement. Refinement of F^2^ against ALL reflections. The weighted R-factor wR and goodness of fit S are based on F^2^, conventional R-factors R are based on F, with F set to zero for negative F^2^. The threshold expression of F^2^ > 2sigma(F^2^) is used only for calculating R-factors(gt) etc. and is not relevant to the choice of reflections for refinement. R-factors based on F^2^ are statistically about twice as large as those based on F, and R- factors based on ALL data will be even larger.

### Fractional atomic coordinates and isotropic or equivalent isotropic displacement parameters (Å^2^)

**Table:**

	*x*	*y*	*z*	*U*_iso_*/*U*_eq_	
S1	0.19580 (2)	0.56351 (4)	0.11644 (2)	0.02585 (12)	
F1	−0.06671 (7)	0.55451 (15)	0.39156 (8)	0.0526 (3)	
O1	0.30601 (7)	0.43713 (13)	0.37816 (7)	0.0286 (2)	
O2	0.12420 (7)	0.65463 (14)	0.12504 (8)	0.0355 (3)	
O3	0.25519 (7)	0.63717 (14)	0.07757 (8)	0.0337 (3)	
C1	0.26010 (9)	0.49468 (18)	0.22631 (10)	0.0250 (3)	
C2	0.35310 (10)	0.45605 (17)	0.25534 (10)	0.0254 (3)	
C3	0.41687 (10)	0.44682 (18)	0.21347 (10)	0.0271 (3)	
H3	0.4025	0.4723	0.1498	0.032*	
C4	0.50184 (10)	0.39963 (18)	0.26662 (11)	0.0288 (3)	
C5	0.52195 (10)	0.3651 (2)	0.36117 (11)	0.0336 (4)	
H5	0.5802	0.3333	0.3967	0.040*	
C6	0.46071 (10)	0.3755 (2)	0.40410 (11)	0.0330 (3)	
H6	0.4751	0.3522	0.4680	0.040*	
C7	0.37701 (10)	0.42184 (18)	0.34923 (10)	0.0269 (3)	
C8	0.23549 (10)	0.48094 (18)	0.30212 (10)	0.0262 (3)	
C9	0.57531 (10)	0.38349 (19)	0.22780 (11)	0.0311 (3)	
H9	0.6053	0.2810	0.2506	0.037*	
C10	0.54480 (11)	0.3797 (3)	0.12306 (12)	0.0440 (4)	
H10A	0.5145	0.4796	0.0978	0.053*	
H10B	0.5022	0.2927	0.0996	0.053*	
C11	0.62254 (12)	0.3564 (3)	0.08978 (14)	0.0464 (5)	
H11A	0.6502	0.2530	0.1113	0.056*	
H11B	0.6010	0.3567	0.0212	0.056*	
C12	0.69027 (13)	0.4850 (3)	0.12594 (15)	0.0476 (5)	
H12A	0.6644	0.5871	0.0989	0.057*	
H12B	0.7413	0.4637	0.1064	0.057*	
C13	0.72119 (13)	0.4951 (3)	0.23060 (16)	0.0555 (5)	
H13A	0.7613	0.5862	0.2518	0.067*	
H13B	0.7546	0.3988	0.2576	0.067*	
C14	0.64422 (12)	0.5131 (2)	0.26502 (13)	0.0426 (4)	
H14A	0.6161	0.6168	0.2454	0.051*	
H14B	0.6666	0.5100	0.3336	0.051*	
C15	0.15393 (10)	0.50362 (18)	0.32210 (10)	0.0274 (3)	
C16	0.07583 (11)	0.4297 (2)	0.26997 (11)	0.0357 (4)	
H16	0.0737	0.3676	0.2180	0.043*	
C17	0.00085 (11)	0.4459 (2)	0.29324 (12)	0.0400 (4)	
H17	−0.0527	0.3954	0.2580	0.048*	
C18	0.00630 (11)	0.5370 (2)	0.36879 (12)	0.0360 (4)	
C19	0.08251 (12)	0.6103 (2)	0.42288 (12)	0.0367 (4)	
H19	0.0841	0.6712	0.4752	0.044*	
C20	0.15689 (11)	0.5932 (2)	0.39912 (11)	0.0320 (3)	
H20	0.2103	0.6428	0.4355	0.038*	
C21	0.14993 (10)	0.3924 (2)	0.04907 (11)	0.0321 (3)	
H21	0.1114	0.3382	0.0783	0.039*	
C22	0.09234 (11)	0.4450 (2)	−0.04767 (11)	0.0408 (4)	
H22A	0.1294	0.4911	−0.0795	0.061*	
H22B	0.0499	0.5237	−0.0429	0.061*	
H22C	0.0606	0.3541	−0.0828	0.061*	
C23	0.22097 (13)	0.2779 (2)	0.04729 (13)	0.0449 (4)	
H23A	0.1936	0.1838	0.0123	0.067*	
H23B	0.2565	0.2482	0.1108	0.067*	
H23C	0.2588	0.3278	0.0176	0.067*	

### Atomic displacement parameters (Å^2^)

**Table:**

	*U*^11^	*U*^22^	*U*^33^	*U*^12^	*U*^13^	*U*^23^
S1	0.02457 (19)	0.0289 (2)	0.02323 (19)	0.00035 (14)	0.00727 (14)	0.00259 (14)
F1	0.0401 (6)	0.0691 (8)	0.0599 (7)	0.0072 (5)	0.0317 (5)	−0.0005 (6)
O1	0.0284 (5)	0.0351 (6)	0.0225 (5)	0.0022 (4)	0.0092 (4)	0.0014 (4)
O2	0.0306 (6)	0.0400 (6)	0.0352 (6)	0.0087 (5)	0.0105 (5)	0.0045 (5)
O3	0.0323 (6)	0.0383 (6)	0.0308 (6)	−0.0037 (5)	0.0114 (5)	0.0073 (5)
C1	0.0246 (7)	0.0265 (7)	0.0239 (7)	−0.0002 (6)	0.0083 (5)	−0.0001 (6)
C2	0.0259 (7)	0.0245 (7)	0.0237 (7)	−0.0011 (5)	0.0060 (6)	−0.0024 (6)
C3	0.0274 (7)	0.0289 (7)	0.0245 (7)	−0.0022 (6)	0.0085 (6)	−0.0026 (6)
C4	0.0254 (7)	0.0289 (7)	0.0309 (8)	−0.0024 (6)	0.0085 (6)	−0.0040 (6)
C5	0.0262 (7)	0.0378 (9)	0.0314 (8)	0.0009 (6)	0.0035 (6)	−0.0015 (7)
C6	0.0328 (8)	0.0373 (9)	0.0246 (7)	0.0018 (7)	0.0047 (6)	0.0008 (6)
C7	0.0276 (7)	0.0278 (7)	0.0259 (7)	−0.0010 (6)	0.0099 (6)	−0.0027 (6)
C8	0.0276 (7)	0.0255 (7)	0.0243 (7)	−0.0002 (6)	0.0075 (6)	−0.0010 (6)
C9	0.0269 (7)	0.0274 (8)	0.0387 (8)	0.0003 (6)	0.0109 (6)	0.0009 (7)
C10	0.0317 (8)	0.0627 (12)	0.0408 (9)	−0.0071 (8)	0.0168 (7)	−0.0154 (9)
C11	0.0415 (10)	0.0555 (12)	0.0514 (11)	−0.0043 (9)	0.0278 (8)	−0.0123 (9)
C12	0.0436 (10)	0.0474 (11)	0.0614 (12)	−0.0017 (8)	0.0304 (9)	0.0042 (10)
C13	0.0354 (10)	0.0700 (14)	0.0628 (13)	−0.0184 (10)	0.0193 (9)	−0.0085 (11)
C14	0.0360 (9)	0.0482 (10)	0.0440 (10)	−0.0148 (8)	0.0143 (8)	−0.0112 (8)
C15	0.0305 (7)	0.0289 (7)	0.0246 (7)	0.0011 (6)	0.0122 (6)	0.0019 (6)
C16	0.0345 (8)	0.0464 (10)	0.0280 (8)	−0.0034 (7)	0.0132 (7)	−0.0063 (7)
C17	0.0307 (8)	0.0544 (11)	0.0346 (9)	−0.0046 (8)	0.0110 (7)	−0.0023 (8)
C18	0.0333 (8)	0.0422 (9)	0.0387 (9)	0.0074 (7)	0.0204 (7)	0.0064 (7)
C19	0.0444 (9)	0.0350 (9)	0.0370 (9)	0.0030 (7)	0.0223 (7)	−0.0034 (7)
C20	0.0343 (8)	0.0330 (8)	0.0304 (8)	−0.0023 (7)	0.0135 (6)	−0.0033 (7)
C21	0.0332 (8)	0.0369 (8)	0.0255 (7)	−0.0078 (7)	0.0094 (6)	−0.0034 (7)
C22	0.0298 (8)	0.0606 (12)	0.0275 (8)	−0.0037 (8)	0.0044 (6)	−0.0036 (8)
C23	0.0535 (11)	0.0387 (10)	0.0366 (9)	0.0040 (8)	0.0084 (8)	−0.0087 (8)

### Geometric parameters (Å, °)

**Table:**

S1—O2	1.4391 (11)	C11—H11B	0.9900
S1—O3	1.4417 (11)	C12—C13	1.515 (3)
S1—C1	1.7498 (15)	C12—H12A	0.9900
S1—C21	1.7910 (16)	C12—H12B	0.9900
F1—C18	1.3545 (18)	C13—C14	1.524 (3)
O1—C8	1.3722 (17)	C13—H13A	0.9900
O1—C7	1.3774 (17)	C13—H13B	0.9900
C1—C8	1.364 (2)	C14—H14A	0.9900
C1—C2	1.452 (2)	C14—H14B	0.9900
C2—C7	1.391 (2)	C15—C16	1.390 (2)
C2—C3	1.398 (2)	C15—C20	1.396 (2)
C3—C4	1.393 (2)	C16—C17	1.388 (2)
C3—H3	0.9500	C16—H16	0.9500
C4—C5	1.408 (2)	C17—C18	1.375 (3)
C4—C9	1.513 (2)	C17—H17	0.9500
C5—C6	1.374 (2)	C18—C19	1.375 (3)
C5—H5	0.9500	C19—C20	1.384 (2)
C6—C7	1.382 (2)	C19—H19	0.9500
C6—H6	0.9500	C20—H20	0.9500
C8—C15	1.471 (2)	C21—C23	1.515 (2)
C9—C10	1.514 (2)	C21—C22	1.524 (2)
C9—C14	1.531 (2)	C21—H21	1.0000
C9—H9	1.0000	C22—H22A	0.9800
C10—C11	1.530 (2)	C22—H22B	0.9800
C10—H10A	0.9900	C22—H22C	0.9800
C10—H10B	0.9900	C23—H23A	0.9800
C11—C12	1.511 (3)	C23—H23B	0.9800
C11—H11A	0.9900	C23—H23C	0.9800
			
O2—S1—O3	118.75 (7)	C11—C12—H12B	109.4
O2—S1—C1	108.56 (7)	C13—C12—H12B	109.4
O3—S1—C1	106.75 (7)	H12A—C12—H12B	108.0
O2—S1—C21	107.78 (7)	C12—C13—C14	111.65 (17)
O3—S1—C21	108.32 (7)	C12—C13—H13A	109.3
C1—S1—C21	106.00 (7)	C14—C13—H13A	109.3
C8—O1—C7	106.89 (11)	C12—C13—H13B	109.3
C8—C1—C2	107.39 (13)	C14—C13—H13B	109.3
C8—C1—S1	127.10 (12)	H13A—C13—H13B	108.0
C2—C1—S1	125.27 (11)	C13—C14—C9	112.12 (16)
C7—C2—C3	119.02 (13)	C13—C14—H14A	109.2
C7—C2—C1	104.49 (13)	C9—C14—H14A	109.2
C3—C2—C1	136.49 (14)	C13—C14—H14B	109.2
C4—C3—C2	118.89 (14)	C9—C14—H14B	109.2
C4—C3—H3	120.6	H14A—C14—H14B	107.9
C2—C3—H3	120.6	C16—C15—C20	119.46 (14)
C3—C4—C5	119.52 (14)	C16—C15—C8	121.58 (14)
C3—C4—C9	123.13 (14)	C20—C15—C8	118.82 (14)
C5—C4—C9	117.35 (14)	C17—C16—C15	120.57 (15)
C6—C5—C4	122.60 (14)	C17—C16—H16	119.7
C6—C5—H5	118.7	C15—C16—H16	119.7
C4—C5—H5	118.7	C18—C17—C16	118.08 (16)
C5—C6—C7	116.35 (14)	C18—C17—H17	121.0
C5—C6—H6	121.8	C16—C17—H17	121.0
C7—C6—H6	121.8	F1—C18—C17	118.50 (16)
O1—C7—C6	125.56 (14)	F1—C18—C19	118.33 (15)
O1—C7—C2	110.82 (13)	C17—C18—C19	123.17 (16)
C6—C7—C2	123.60 (14)	C18—C19—C20	118.24 (15)
C1—C8—O1	110.41 (13)	C18—C19—H19	120.9
C1—C8—C15	136.08 (14)	C20—C19—H19	120.9
O1—C8—C15	113.50 (12)	C19—C20—C15	120.46 (15)
C4—C9—C10	114.42 (13)	C19—C20—H20	119.8
C4—C9—C14	111.02 (14)	C15—C20—H20	119.8
C10—C9—C14	109.81 (15)	C23—C21—C22	112.41 (14)
C4—C9—H9	107.1	C23—C21—S1	111.55 (11)
C10—C9—H9	107.1	C22—C21—S1	108.49 (12)
C14—C9—H9	107.1	C23—C21—H21	108.1
C9—C10—C11	111.03 (15)	C22—C21—H21	108.1
C9—C10—H10A	109.4	S1—C21—H21	108.1
C11—C10—H10A	109.4	C21—C22—H22A	109.5
C9—C10—H10B	109.4	C21—C22—H22B	109.5
C11—C10—H10B	109.4	H22A—C22—H22B	109.5
H10A—C10—H10B	108.0	C21—C22—H22C	109.5
C12—C11—C10	111.13 (16)	H22A—C22—H22C	109.5
C12—C11—H11A	109.4	H22B—C22—H22C	109.5
C10—C11—H11A	109.4	C21—C23—H23A	109.5
C12—C11—H11B	109.4	C21—C23—H23B	109.5
C10—C11—H11B	109.4	H23A—C23—H23B	109.5
H11A—C11—H11B	108.0	C21—C23—H23C	109.5
C11—C12—C13	111.19 (16)	H23A—C23—H23C	109.5
C11—C12—H12A	109.4	H23B—C23—H23C	109.5
C13—C12—H12A	109.4		
			
O2—S1—C1—C8	20.02 (16)	C5—C4—C9—C10	164.58 (16)
O3—S1—C1—C8	149.13 (14)	C3—C4—C9—C14	109.38 (17)
C21—S1—C1—C8	−95.53 (15)	C5—C4—C9—C14	−70.45 (19)
O2—S1—C1—C2	−153.56 (13)	C4—C9—C10—C11	−177.68 (15)
O3—S1—C1—C2	−24.45 (15)	C14—C9—C10—C11	56.7 (2)
C21—S1—C1—C2	90.89 (14)	C9—C10—C11—C12	−57.9 (2)
C8—C1—C2—C7	−0.18 (17)	C10—C11—C12—C13	55.8 (2)
S1—C1—C2—C7	174.46 (11)	C11—C12—C13—C14	−53.9 (2)
C8—C1—C2—C3	179.77 (17)	C12—C13—C14—C9	54.0 (2)
S1—C1—C2—C3	−5.6 (3)	C4—C9—C14—C13	177.30 (16)
C7—C2—C3—C4	1.7 (2)	C10—C9—C14—C13	−55.2 (2)
C1—C2—C3—C4	−178.29 (16)	C1—C8—C15—C16	53.2 (3)
C2—C3—C4—C5	−1.1 (2)	O1—C8—C15—C16	−128.01 (16)
C2—C3—C4—C9	179.09 (14)	C1—C8—C15—C20	−131.11 (19)
C3—C4—C5—C6	0.1 (3)	O1—C8—C15—C20	47.7 (2)
C9—C4—C5—C6	179.98 (15)	C20—C15—C16—C17	0.8 (3)
C4—C5—C6—C7	0.2 (3)	C8—C15—C16—C17	176.42 (16)
C8—O1—C7—C6	−178.35 (15)	C15—C16—C17—C18	0.2 (3)
C8—O1—C7—C2	0.27 (17)	C16—C17—C18—F1	179.46 (16)
C5—C6—C7—O1	178.87 (15)	C16—C17—C18—C19	−1.1 (3)
C5—C6—C7—C2	0.4 (2)	F1—C18—C19—C20	−179.56 (15)
C3—C2—C7—O1	179.99 (13)	C17—C18—C19—C20	1.0 (3)
C1—C2—C7—O1	−0.06 (17)	C18—C19—C20—C15	0.0 (3)
C3—C2—C7—C6	−1.4 (2)	C16—C15—C20—C19	−0.9 (2)
C1—C2—C7—C6	178.59 (15)	C8—C15—C20—C19	−176.63 (15)
C2—C1—C8—O1	0.36 (17)	O2—S1—C21—C23	−172.18 (12)
S1—C1—C8—O1	−174.15 (11)	O3—S1—C21—C23	58.15 (14)
C2—C1—C8—C15	179.15 (17)	C1—S1—C21—C23	−56.11 (14)
S1—C1—C8—C15	4.6 (3)	O2—S1—C21—C22	63.47 (12)
C7—O1—C8—C1	−0.39 (17)	O3—S1—C21—C22	−66.20 (12)
C7—O1—C8—C15	−179.48 (12)	C1—S1—C21—C22	179.55 (11)
C3—C4—C9—C10	−15.6 (2)		

### Hydrogen-bond geometry (Å, °)

**Table:**

Cg is the centroid of the C2–C7 benzene ring.

**Table:**

*D*—H···*A*	*D*—H	H···*A*	*D*···*A*	*D*—H···*A*
C14—H14A···Cg^i^	0.99	2.68	3.667 (2)	172

Symmetry codes: (i) −*x*+1, *y*+1/2, −*z*+1/2.
